# Sex-related differences in adult attention-deficit hyperactivity disorder patients – An analysis of external globus pallidus functional connectivity in resting-state functional MRI

**DOI:** 10.3389/fpsyt.2022.962911

**Published:** 2022-09-02

**Authors:** Gabriele Dupont, Daan van Rooij, Jan K. Buitelaar, Andreas Reif, Oliver Grimm

**Affiliations:** ^1^Department of Psychiatry, Psychosomatic Medicine and Psychotherapy, University Hospital, Goethe University Frankfurt, Frankfurt, Germany; ^2^Department of Cognitive Neuroscience, Donders Institute for Brain, Cognition, and Behavior, Donders Center for Cognitive Neuroimaging, Radboud University Medical Center, Nijmegen, Netherlands

**Keywords:** ADHD, resting state fMRI, gender medicine, ADHD comorbidities, ADHD sex-differences

## Abstract

In the last two decades, there has been a growing body of research that identified sex-related differences in attention-deficit hyperactivity disorder (ADHD). Our objective was to quantify whether these sex differences are based on altered functional brain connectivity profiles. In addition, we investigated whether the presence of comorbid disorders, including depression, substance use disorder (SUD) and overweight, influenced these sex differences. A seed-based connectivity analysis of the external globus pallidus (GPe), an important inhibitory relay hub of the fronto-thalamo-striatal-loop, was performed. In a first step, we searched for sex-related differences in ADHD patients (*N* = 137) and separately in healthy controls (HC) (*N* = 45), after that, we compared an equal group of HC and ADHD patients to compare sex-related differences in ADHD patients and HC. In a second step, we studied whether the neural basis of comorbidity patterns is different between male and female patients. We observed that male ADHD patients demonstrated a decrease in functional connectivity (FC) from the GPe to the left middle temporal gyrus compared to female ADHD patients. Moreover, within the full ADHD group (*N* = 137), there was a lower FC in male patients from GPe to the right frontal pole/middle frontal gyrus compared to female patients. Male ADHD patients with depression demonstrated decreased FC from the GPe to parts of the occipital cortex compared to female ADHD patients with depression. No such effect was demonstrated for overweight or SUD. The current study reveals different FC profiles in males and females with ADHD, which are centered around altered connectivity with the GPe. An improved understanding of sex-differences in ADHD, and the role of comorbid disorders, therein can result in improved diagnostic and therapeutic opportunities for ADHD patients.

## Introduction

Attention-deficit hyperactivity disorder (ADHD) is a neurodevelopmental disorder with cardinal symptoms of inattentiveness, hyperactivity, and impulsiveness, which leads to impairments in everyday life. ADHD manifests in childhood, but persists in a substantial number of the cases into adulthood (1). However, some of these symptoms are not stable across the lifespan, e.g., attention problems are much more persistent in adulthood than hyperactivity (2, 3). The developmental trajectory of ADHD patients shows a substantial occurrence of comorbid disorders such as addiction and depression (4). Over the last two decades, there has been a growing body of research, which has established a male preponderance in childhood ADHD, whereas the gender balance in adulthood tends to be equal (5).

Previously, in Biederman et al. reported a sex-by-ADHD interaction in the association between ADHD and SUD in referred children, with the strongest association being observed in girls (6). Girls with ADHD were also at significantly greater risk for co-morbid major depression than girls without ADHD, but had a significantly lower rate of comorbid major depression than boys with ADHD (6). Few previous studies have evaluated sex differences in comorbidity among adults with ADHD, and the results of existing studies are sometimes contradictory. A study from 2016, with a large clinical referral sample of adult ADHD, did not find sex differences in ADHD prevalence, psychosocial impairment, or number of comorbidities, although the specific comorbid diagnoses were sex dependent (7). Females compared with males presented higher rates of mood disorders in general and major depression in particular (7). In contrast to this, men diagnosed with ADHD were more likely than women to develop SUD in general (7). In a large population-based study of adults with ADHD, prevalence differences associated with ADHD were significantly greater in women for anxiety, depression, bipolar disorder, and personality disorders, whereas findings were significantly greater in men for schizophrenia and substance use disorders (SUD) (8). However, more meaningful than prevalence differences are prevalence ratios which account for baseline sex differences. Sex differences in prevalence ratios are stronger for males than females for anxiety disorders and depression, suggesting that adult ADHD increases the risk toward depression in men more than in women (Hartman et al., in preparation). In summary, there are clear epidemiological indications that, not only ADHD, but also associated comorbid disorders, appear to be different between men and women (8, 9).

The available data purports that the pathogenesis of ADHD is linked to a dysregulation of impulse control. One possible neuroanatomical pathway underlying this, is that of the indirect inhibitory fronto-basal pathways involving the dorsolateral prefrontal cortex (DLPFC), caudate, putamen, globus pallidus (GP), thalamus, and motor areas (10, 11). In the present study, we concentrated on a neuroanatomical hub central to this pathway, namely, the external globus pallidus (GPe). The internal globus pallidus (GPi) is inhibited by the external GP, which leads to inhibition of the thalamus, and in turn inhibits the motor loop (12, 13). The GP has many important roles in the representation of a reward-associated signal: most pertinently for this study, it has been shown that the GP mediates a reward signal to the habenula and that valence encoding in the GP shows an interaction between DA blockade and magnitude of reward (14). This information is relayed to the substantia nigra/area ventralis tegmentalis (VTA) area–a central circuit of the reward response. Thus, GPe/GPi activation may play a critical role in facilitating inhibition of immediate reward. In animal studies, bicuculline injections into different subregions of the GPe in primates have been shown to elicit behavioral disturbances, including attention deficits and hyperactivity, which raises the possibility of a direct GPe involvement in the symptoms of ADHD (12).

Thus, the literature to date suggests that there are sex differences in ADHD, and especially in cases with comorbid disorders, but these differences are not yet understood. We postulate that the functional neuroanatomy of impulse control plays a critical role, and that the GP is an attractive candidate to investigate such dysregulated functional connectivity (FC). This might enable us to test whether there are sex differences in basic neuronal strategies in resting-state fMRI neuroimaging. Our objectives were therefore twofold. First, we were interested in studying sex-specific differences between ADHD and healthy controls (HC) for GP-based seed connectivity. Secondly, we wanted to verify these sex-specific neural patterns in ADHD-related comorbidities. First, we searched for sex-specific differences in FC in a large ADHD sample of 137 subjects and separately in 45 HC. In a second step, we selected a similar group of healthy subjects and ADHD patients (age, site) to be tested for sex-dependent effects, because the group of ADHD patients were significantly older than the group of HC. Finally, in the sample of 137 ADHD patients we tested whether we could verify sex-specific neural patterns in ADHD-related comorbidities.

## Materials and methods

### Participants

#### Healthy volunteers

Healthy volunteers of both sexes with an age between 18 and 50 years from Germany were included in this study (for demographics see Table 1). Participants had to demonstrate good German language skills and were excluded in case of any severe general or neurological disorders, any history of psychiatric disorders or previous allergic drug response. Taking medication other than thyroid hormone replacement therapy or hormonal contraceptives was an exclusion criterion, as was pregnancy. Patients with MRI contraindications were excluded. Participants were recruited *via* local advertising measures and examined by a registered psychiatrist. A total of 45 healthy volunteers (22M/23F, mean age: 22.81 years, SD: 2.71 years) were included. The average body weight of the subjects included was 72.86 kg (SD: 12.91 kg) with an average height of 1.75 m (SD: 0.11 m), which corresponds to an average BMI of 23.59 (SD: 3.08). The participants received an expense allowance of approx. A total of 50 € for participation in the study.

**TABLE 1:** Demographic overview.

	ADHD	HC	HC (ADHD vs. HC)	ADHD (ADHD vs. HC)
Number of participants	137	45	36	36
Age (years)/SD	31.96 (8.92)	22.81 (2.71)	23.19 (2.82)	24.47 (3.18)
**Site**				
Nijmegen	53	0	0	0
Frankfurt am Main	84	45	36	36
**Sex**				
Female	75	23	18	18
Male	62	22	18	18
**Number of comorbidities**				
1	55	0	0	17
2	41	0	0	6
3	12	0	0	2
**Type of comorbidities**				
Overweight	66	11	10	12
Depression	64	0	0	14
SUD	43	0	0	8
**Subgroups**				
Overweight	25	11	10	6
Depression	17	0	0	7
SUD	13	0	0	4
Overweight and depression	23	0	0	3
Overweight and SUD	6	0	0	1
Depression and SUD	12	0	0	2
Overweight and depression and SUD	12	0	0	2

The demographics and clinical characteristics are given for the connectivity sample. Standard deviations are given in brackets.

SD, standard deviation; SUD, substance use disorder.

The approval to conduct the study was given by the local ethics commission (Faculty of Medicine, University Hospital, Goethe University, Frankfurt am Main) and is subject to the Declaration of Helsinki of the “World Medical Association: Ethical Principles for Medical Research Involving Human Subjects” and the “Guidelines for Good Clinical Practices (GCP).” In addition, the study was registered as a clinical trial in the German study registry under the ID: DRKS00011209. Written informed consent was obtained from each volunteer before the start of the study.

#### Attention-deficit hyperactivity disorder patients

A total of 137 ADHD patients with an age between 18 and 50 years were included in this study (62M/75F, mean age: 31.96 years, SD: 8.92 years) (for demographics see Table 1). Recruitment took place at the Donders Centre for Cognitive Neuroimaging, Nijmegen, Netherlands, and the Goethe University Frankfurt am Main. The average body weight of the subjects included was 78.78 kg (SD: 16.92 kg) with an average height of 1.74 m (SD: 0.09 m), which corresponds to an average BMI of 25.92 (SD: 5.53). Including criteria were sufficient German/Dutch language skills, normal or corrected-to-normal vision, childhood diagnosis of ADHD (diagnosed by a specialist following the DSM-IV-criteria, plus ADHD questionnaires like CAARS, Wender-Utah-Scale) and a chronic course (WURS-k >30). In addition, we included ADHD patients with comorbidities like depression (DSM IV) and SUD (DSM-IV) and/or overweight (BMI >25 kg/m^2^). Exclusion criteria were other mental illnesses (apart from ADHD, depression, and SUD), serious acute or chronic physical diseases, pregnancy, as well as exclusion criteria of the MRI examination. Only patients with at least 4 weeks of stable medication regimen were included. Stimulants, alcohol, and nicotine were stopped on the day of the scan. Patients with antipsychotic medication were excluded. Participants were examined by a registered psychiatrist in Frankfurt in a specialized ADHD-outpatient clinic. In Nijmegen, selection and diagnostic procedures were conducted by trained psychiatrists or psychologists. The project was carried out in accordance with the provisions of the Declaration of Helsinki World Medical Association, (15) and the European guidelines on Good Clinical Practice and was approved by the Ethics Committee of the Medical Faculty of the J.W. Goethe University Frankfurt am Main (reg. no. 256/16) and in Nijmegen by the Radboud University (reg. no. ABR64162). The study was registered as a clinical trial in the German study registry under the ID: DRKS00011248. The subjects received 10€ per hour for participation.

### Image acquisition

Participants underwent MRI scans on a 3 Tesla full body MR scanner (Siemens Magnetom Trio syngo MR A35) at the Brain Imaging Center in Frankfurt am Main and a 3 T MR scanner (Siemens PRISMA) in Nijmegen to obtain high resolution structural images and resting state functional MRI (rs-fMRI) images.

### Pre-processing

Images of 182 participants underwent a preprocessing algorithm done with the SPM-based CONN-toolbox V 18.b. to minimize the effects of unwanted variability in the blood oxygenation level-dependent (BOLD) signal (16). The images were realigned, slice-time corrected, spatially normalized to standard stereotactic space (Montreal Neurological Institute [MNI] template), resampled to 3 mm isotropic voxels, and smoothed with 8 mm full-width at half maximum Gaussian kernel (16). After the functional data has been preprocessed, the BOLD signal often still contains a considerable amount of noise which are minimized by CONN’s default denoising pipeline (16). A band-pass filter (0.01–0.1 Hz) was used to suppress non-neural signals. Additional noise correction was performed by regressing the motion parameters obtained from the realignment procedure and the first order derivative of the motion parameters. We generated QA plots of mean motion, maximum motion, and the maximum and mean GS change. QA plots of the motion parameters can be found in the Supplementary Figures 2, 3. CONN’s denoising pipeline defines 12 potential noise components from the estimated subject-motion parameters (16, 17) and uses scrubbing to remove any influence of the identified outlier scans to reduce motion related BOLD variability (16, 18). Signals from the cerebrospinal-fluid and white-matter were regressed with a component base noise reduction method (CompCor). This method takes the principal components of white matter/cerebrospinal fluid regions as nuisance regressors (19) and can avoid the global-scaling related anticorrelation issues with a higher specificity and sensitivity for positive correlations (20). We used a seed-based connectivity analysis using subcortical seed region-of-interest (ROI), comprising brain regions that are central to the meso-limbic system, known to be involved in reward processing and were reliably defined in the OTI Atlas of Pauli et al. (21). This atlas was constructed based on high- spatial resolution T1- and T2-weighted structural images from 168 young adults (21). Tissue boundaries were used to delineate subcortical nuclei which were combined to form a probabilistic atlas (21). Out of the atlas’ parcellated regions, we chose the external segments of the GP.

### Data analysis: Group statistics

First-level correlation maps were calculated by extracting the residual BOLD-time course from the ROI seeds and correlating these with the other voxels within the brain. These correlation coefficient maps were then converted into a normally distributed z score (Fisher transformation). Transformed correlation maps were used for multiple regression tests and 2 × 2 between-subjects ANOVA interaction. In a first step we performed a multiple regression test to analyze the influence of sex in the full ADHD group (*N* = 137). Age and site (Nijmegen/Frankfurt) were included as covariates in the model to account for age and site-related variability between sub-groups of interest. Separately we performed a multiple regression test to analyze the influence of sex in a group of *N* = 45 HC with age as covariate. In a second step we chose a group of 72 probands: 36 HC (18F; mean age 23.19; SD: 2.82) and 36 ADHD patients (18F; mean age: 24.47; SD: 3.18) out of study sample to compare sex-related differences in ADHD patients and HC. We matched the ADHD patients and HC by sex, age, and location, since the fMRIs of the HC were performed only in Frankfurt and they were significantly younger than the ADHD patients. We performed matching manually, with the rater blinded to the results of the first- and second-level connectivity analysis of *N* = 137 ADHD patients and *N* = 45 HC. In the end, we were able to form a more homogeneous group consisting of 36 HC (18F; mean age 23.19; SD: 2.82) and 36 ADHD patients (18F; mean age: 24.47; SD: 3.18) with a lower SD in ADHD patients compared to the group of *N* = 137 ADHD subjects (62M/75F, mean age: 31.96 years, SD: 8.92 years).

We explored the effect of sex by using a between-subjects 2 × 2 ANOVA with four groups (Male ADHD vs. Female ADHD vs. Male HC vs. Female HC). To explore whether the neural basis of comorbidity patterns is different between male and female patients, we used three between-subjects ANOVA tests to study separately the interaction of sex and comorbid depression (Male ADHD patients + depression vs. Female ADHD patients + depression vs. Male ADHD patients without depression vs. Female ADHD patients without depression), sex/SUD and sex/overweight. For correction of multiple testing during second-level statistics we used cluster-wise whole-brain analysis which uses a combination of an uncorrected *p* < 0.001 height threshold to initially define clusters of interest from the original statistical parametric maps, and a FDR- corrected *p* < 0.05 cluster-level threshold to select the significant clusters among the resulting clusters.

## Results

### Connectivity main effects

To validate our seed-based connectivity approach we calculated seed-maps to illustrate the connectivity’s main effect in HC and ADHD patients. The GPe in both, HC and ADHD, showed connectivity with other parts of the basal ganglia and parts of the frontal- (precentral gyrus, cingulate gyrus, frontal orbital cortex, frontal pole, middle frontal gyrus…) temporal (middle temporal gyrus, temporal pole, inferior temporal gyrus…), parietal (post-central gyrus…) and occipital cortex (occipital fusiform cortex, lateral occipital cortex, occipital pole, lingual gyrus) among others. A detailed overview is given in the Supplementary Figure 1.

### Sex-related differences in a large attention-deficit hyperactivity disorder sample

**FIGURE 1:**
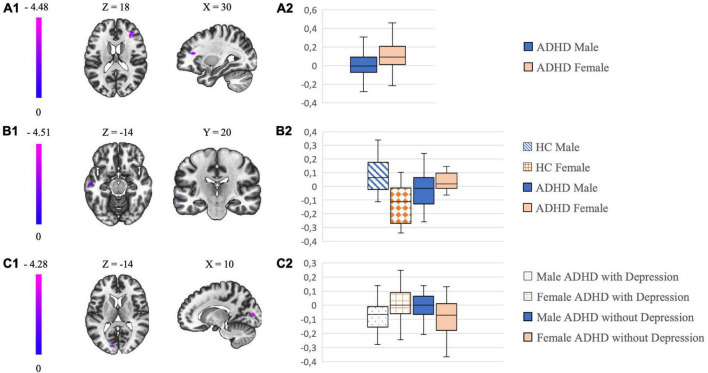
The left column shows in (A1–C1) the most significant clusters of the different connectivity analysis and their localization. The color-coding bar depicts the voxel-wise *T*-values. The column on the right with box plots in (A2–C2) show the mean beta values extracted from the respective cluster which is illustrated on the left.

In the whole ADHD group of 137 patients we found differences in FC (details are given in Table 2). With the external GPe as seed, male patients with ADHD showed a lower FC to the frontal pole/middle frontal gyrus right compared to females with ADHD (details are given in Figure 1).

**TABLE 2:** Significant clusters from the seed-region mask of the GPe.

**Effect of sex in ADHD, female >male, two-sided**						
Seed region	Brain region	Cluster	MNI-coordinates			pFDR
			
		size	x	y	z	
GPe	Frontal pole R; Middle frontal gyrus R	225	+ 36	+36	+ 20	0.050
**Effect of sex in HC, male >female, two-sided**						
Seed region	Brain region	Cluster size	MNI-coordinates			pFDR
			
			x	y	z	
GPe	Middle temporal gyrus, posterior division L;	116	−58	−16	−12	0.030
	Middle temporal gyrus, anterior division L					
**ANOVA: sex (female; male) and ADHD (ADHD; HC), two-sided**						
Seed region	Brain region	Cluster	MNI-coordinates			pFDR
			
		size	x	y	z	
GPe	Middle temporal gyrus posterior division L;	111	−64	−20	−14	0.041
	Middle temporal gyrus anterior division L					
**ANOVA: sex (female; male) and depression (yes; no) in ADHD, negative-contrast**						
Seed region	Brain region	Cluster	MNI-coordinates			pFDR
			
		size	x	y	z	
GPe	Lingual Gyrus L/R; Intracalcarine Cortex L/R;	340	−14	−88	+ 10	0.008
	Occipital Pole L; Supracalcarine Cortex R					

The table shows significant cluster, their size in voxel, and their localization in the MNI space as MNI coordination in the order x y z. The threshold for clusters was set at *p* < 0.05, the threshold for voxels was set at *p* < 0.001. GPe, external globus pallidus; L, left; R, right.

### Sex-related differences in attention-deficit hyperactivity disorder vs. healthy controls

When comparing sex-related connectivity differences of HC (*N* = 36) with ADHD patients (*N* = 36) (details are given in Table 2), we noticed a lower FC in males with ADHD from the GPe to the middle temporal gyrus left compared to females with ADHD. This effect was opposite in HC: male HC demonstrated an increase in FC from GPe to the middle temporal gyrus left compared to female HC (details are given in Figure 1). In correlation we searched for sex-related differences in the group of HC (*N* = 45) and found a decrease in FC from the GPe to the middle temporal gyrus left in female HC compared to male HC.

### Interaction between comorbidity and sex in a large attention-deficit hyperactivity disorder sample

In the analysis of sex-related comorbidity patterns there was a significant between group difference in the FC between GPe and parts of the occipital cortex (occipital pole, lingual gyrus, intra-/supracalcarine cortex) (details are given in Table 2): Male ADHD patients with depression demonstrated a decreased FC compared to female ADHD patients with depression, with the opposite effect observed in ADHD patients without depression (details are given in Figure 1). We did not find any significant sex-related differences in FC of the GPe by analyzing the comorbidity patterns of SUD and overweight.

### Movement-related effects

We extracted mean-motion and max-motion values from the ADHD vs. HC analysis (*N* = 72) and performed independent samples tests. Patients and controls differed in motion. HC showed significantly higher mean-motion and max-motion compared to ADHD patients (*p* < 0.001). The mean across the mean-motion FD was 0.05 in ADHD patients with a standard deviation of 0.03, in HC the mean of mean-motion FD was 0.1 with a standard deviation of 0.04. The mean of max-motion in HC was 1.35 with a SD of 0.22 and the mean of max-motion in ADHD was 0.38 with a SD of 0.15. We did not detect any sex-related differences in the group of HC in mean-motion (*p* = 0.17) or max-motion (*p* = 0.53). The average of mean-motion in males was 0.11 with a SD of 0.05 and 0.09 with a SD of 0.03 in females. The mean of max-motion in males was 1.37 with a SD of 0.27 and 1.32 with a SD of 0.16 in female HC. We did not detect any sex-differences of mean-motion (*p* = 0.73) or max-motion (*P* = 0.62) in ADHD patients. The average of mean-motion in males was 0.06 with a SD of 0.03 and 0.05 with a SD of 0.04 in females with ADHD. The mean of max-motion in males was 0.37 with a SD of 0.16 and 0.39 with a SD of 0.15 in females with ADHD. No sex-differences in mean-motion (*p* = 0.28) or max-motion (*p* = 0.94) were demonstrated regardless of group either. From the large ADHD sample (*N* = 137) we calculated additional mean framewise displacements (FD) according to Power et al. (18). Using an independent samples test, no significant sex difference in mean FD could be detected (*p* = 0.17). The average mean FD in males was 0.23 with a SD of 0.12 and 0.26 in females with a SD of 0.14.

## Discussion

Our current study reveals different FC profiles between males and females with ADHD, which are centered around altered connectivity with the GPe. General connectivity changes in ADHD have been documented before, but the direction of these effects remains unclear, with both hyperconnectivity (22–25), as well as hypoconnectivity (26–28), of the fronto-striatal network in patients with ADHD being observed. The GPe is a special module of this reward-related network. Apart from motor function, the GP is thought to integrate cognitive and reward-related information (29, 30), functions that are compromised in ADHD. Basal ganglia regions such as the GPe are also structurally impaired in children with ADHD, but the effect appears to change with age and under stimulant therapy (31, 32). Therefore, our study serves to shed more light on the function of GPe in ADHD patients (with and without comorbidities).

Our first main finding is that females with ADHD showed a higher FC from the GPe to the prefrontal cortex compared to males with ADHD. The prefrontal cortex is involved in executive functions and emotion control: it shapes decision making and affective behavior, social disinhibition, and impulse control (33). The middle frontal gyrus as part of our significant cluster seems to be a key hub of the ventral attention network (VAN), which is thought to be implicated in externally oriented attention (34). Abnormalities in the prefrontostriatal circuit are an important correlate of ADHD and have been well described in multiple studies (10, 11). The GPe is part of the frontal cortico-basal ganglia network and has widespread projections to other basal ganglia nuclei (35). A study using diffusion-weighted MR imaging tractography showed that the GPe is not only indirectly involved, but has direct cortical connections to the prefrontal and orbitofrontal cortex and parts of the parietal and temporal cortex, which leads to an involvement in associative and limbic networks (36).

Prior studies assessing sex differences in ADHD were inconsistent in terms of sex effects as well as the exact nature of the difference: A study by Sörös et al. which included resting state fMRI data sets from 38 adults with ADHD, did not reveal any sex differences at all (37). In contrast, a study of Rosch et al. sex-effects in children with ADHD in resting state fMRI were identified. In more detail, girls with ADHD showed atypical intrinsic FC between the striatum and the prefrontal cortex, including stronger positive FC with the anterior cingulate cortex and a stronger negative FC with the dorsolateral prefrontal cortex (38). These findings of Rosch et al. suggest that fronto-subcortical functional networks are more affected in girls with ADHD (38).

However, our finding of sex-differences in the ADHD group does not answer whether it is specific for ADHD or just a general sex difference. Therefore, we examined general sex differences from the GPe in the group of *N* = 45 HC, then we compared the interaction of the GPe in ADHD and HC. In the HC group, we found a decrease in FC from the GPe to the middle temporal gyrus left in females compared to males. When comparing a smaller sample with ADHD and HC for sex differences, we found an interaction between the GPe, and the middle left temporal gyrus: there was a significant connectivity difference between healthy subjects with a higher connectivity in healthy males compared to healthy females. In ADHD patients, the effect was less pronounced with an opposite direction. Parts of the temporal lobe are thought to underlie the top-down direction of attentional resources during response inhibition (39) and FC abnormalities in resting state, task-based fMRI studies or PET-studies of ADHD patients are already documented in these regions (11, 39–41). Importantly, the connection between GPe and the temporal lobe is part of the associative network of GPe connectivity (29).

We cannot assume specificity for the effect in the large ADHD sample. Patients with ADHD show sex differences in connectivity between the external GPe and the right frontal pole/middle frontal gyrus. Whether the effect is specific for ADHD cannot be finally clarified based on the analyses. However, comparing the results of the first three analyses, one direction of the sex-effect emerges. Connectivity between the GPe, frontal and temporal brain areas appear to be stronger in ADHD females compared to males, with the sex-effect being reversed and more pronounced in healthy subjects. This suggests that in patients with ADHD there is a loss of sex-specialization in GPe-connectivity.

Since comorbid disorders play a major role in the negative trajectory of (adult) ADHD, we investigated a sex*comorbidity (SUD, overweight and depression) interaction. No such effect was demonstrated for overweight or SUD. However, male ADHD patients with depression demonstrated decreased FC between the GPe and the occipital cortex compared to female ADHD patients with depression, with the opposite effects observed in ADHD patients without depression. Taken together, these findings suggest an ADHD-specific attentional defect mediated by the GPe-occipital cortex FC: The occipital cortex forms part of the dorsal attentional network, which maintains attention and suppress stimuli that are irrelevant (11). Indeed, recent neuroimaging studies demonstrated an involvement of the occipital cortex in ADHD (11 – 42). A reduction of gray matter volume in the visual cortex of adults with ADHD (42), and a decreased cortical thickness in medial occipital cortex were detected. In task-based functional studies, children with ADHD showed deactivation of parietal and occipital regions during spatial tasks, whereas adults with ADHD showed occipital hyperactivation during inhibition, working memory and attentional tasks (11).

Interestingly, this is not specific for ADHD as involvement of the occipital cortex has also been described in depression. In a study of resting state fMRI, major depression was linked to a decrease in FC between the ventral attention network and regions of precuneus extending to occipital and posterior cingulate cortex, regions which are involved in visual attention (43). In a task-based fMRI study of Kaiser et al. the impact of depression on visual and prefrontal cortical activity, as well as their connectivity during visual working memory, were examined (43). How ADHD pathophysiology is linked to these FC changes and subsequently depression, is as yet unclear. Maturational deficits in fronto-striatal pathways might have a role, which however needs to be empirically tested.

In summary, both disorders, ADHD and depression, compromise attention processing, including visual attention and working memory. There have been very few studies exploring the effect of sex and comorbidities in ADHD with resting state fMRI analysis. One study is a rsfMRI study of Park et al. where several regions linked with depression and anxiety were identified by using graph-theoretic network measures: middle frontal gyrus, superior parietal lobule, middle temporal gyrus, and middle occipital gyrus were identified as regions that displayed sex differences and a strong correlation with DSM scores only in the female ADHD group (44). Like mentioned in the introduction, while the absolute risk for depression in female ADHD patients is higher than in male ADHD patients, the relative risk of depression might be equal with a higher tendency in females comparing the existing literature (7–9, 45). Further investigations need to analyze the possible link between sex and depression in ADHD patients and the involvement of the GPe and the occipital cortex as it is a region that is compromised in both diseases.

Our results support the hypothesis that ADHD affects males and females differently as we found less sex-specific differentiation in ADHD patients. We cannot say to what extent our results have a direct influence on differences in the clinical manifestation of ADHD. However, future investigations to correlate the connectivity differences with clinical questionnaires may be a solution.

In our motion analysis we did not detect any sex-differences. Surprisingly, the healthy subjects moved significantly more than the ADHD patients, detected in both mean-motion and max-motion. Nevertheless, all our participants showed low and acceptable mean motion. The small but significant difference of mean- and max-motion between HC and patients might come from the fact that almost all HC participants had no prior MRI experience, while the patient group had more MRI experience.

While our study has several strengths, such as large sample size in our first analysis, we nevertheless would like to point out some limitations. While we describe connectivity patterns of the GPe, we are aware that rs-fMRI in our technical setting might not be optimal in delineating the neuroanatomical different nuclei reliably. Moreover, resting state fMRI is not directly linked to a behavioral output. Future studies should complement our analysis with task-based behavioral paradigms linked to ADHD pathophysiology like reward anticipation- or verbal working memory tasks. The sample sizes of the second (*N* = 45) and third (*N* = 72) analysis are smaller comparing to our first analysis of *N* = 137 ADHD patients. The reproducibility of studies with small samples has recently been criticized by Marek et al. (46). The results need to be replicated in a larger sample, so we aim for a larger sample in future studies and would report the smaller sample as a limitation. Furthermore, we did not correlate more fine-grained dimensional scales with our findings. Connectivity patterns might change between adolescence and adulthood and might lead to sex-specific comorbidity patterns, as well a sex-specific neuronal strategies. In further studies it would be important to include children and adolescents to investigate developmental trajectories relating to our main readouts.

## Conclusion

To our knowledge, this is the first study to examine sex-specific FC networks using a seed-based connectivity analysis of the external GPe in adult ADHD patients with and without comorbidities. The study serves to improve our knowledge of the involvement of the GPe in ADHD and the sex-specific recruitment of this network. When comparing subjects with ADHD and HC we observed an interaction between the GPe and the middle left temporal gyrus with a more pronounced effect in HC. Within the analysis of the large ADHD sample, an interaction between the GPe and the frontal pole/middle frontal gyrus right could be identified. The direction of the sex effect was more pronounced than in the analysis between ADHD patients and HC, with the result that females with ADHD showed a higher connectivity between the GPe and the frontal pole/middle right frontal gyrus. The results suggests that in patients with ADHD there is a loss of sex-specialization in GPe-connectivity. Males with ADHD and depression demonstrated a decreased FC between the GPe and parts of the occipital cortex compared to females with ADHD and depression. Taken as a whole, this study contributes to our understanding of the neurobiological correlates of ADHD and suggests possible differences between males and females with ADHD centered on altered connectivity with the GPe, helping to provide a different perspective on current research and new ideas for further studies.

## Data availability statement

The raw data supporting the conclusions of this article will be made available by the authors, without undue reservation.

## Ethics statement

The studies involving human participants were reviewed and approved by the Local Ethics Commission (Faculty of Medicine, University Hospital, Goethe University, Frankfurt am Main). The patients/participants provided their written informed consent to participate in this study.

## Author contributions

OG, JB, DR, and AR were involved in the conception and design of the study. GD conducted the data collection with the help of DR. GD analyzed the data including the pre-processing steps and wrote the manuscript. GD interpreted the results with the help of OG. All authors contributed to the revision of the manuscript, read and approved the submitted version.
