# Supplementary figures and images for: Sex-related differences in adult attention-deficit hyperactivity disorder patients – An analysis of external globus pallidus functional connectivity in resting-state functional MRI
Source: Front Psychiatry. 2022 Sep 2;13:962911. doi: 10.3389/fpsyt.2022.962911 (PMC9478108; doi:10.3389/fpsyt.2022.962911)

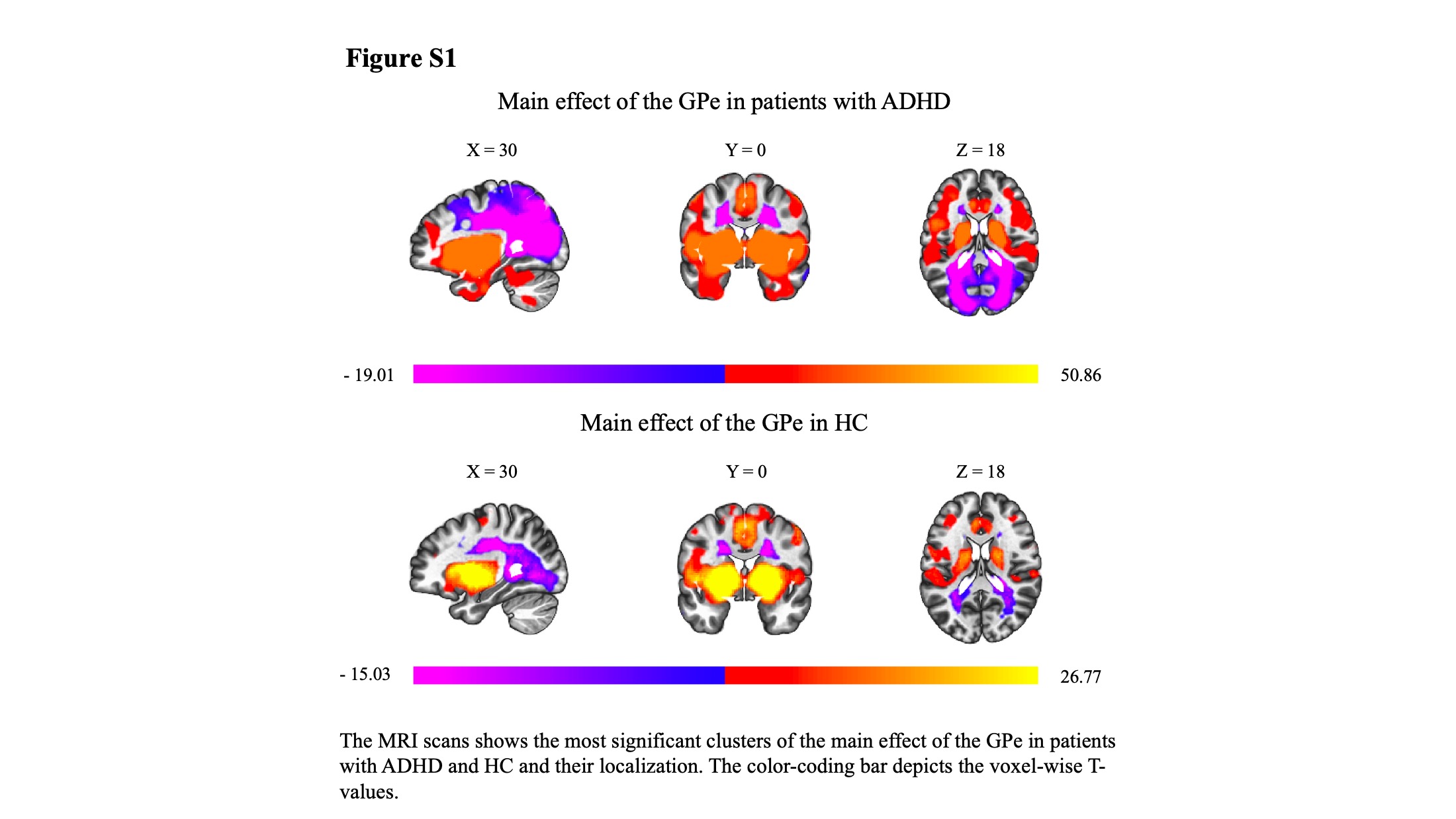

Supplement: Supplementary file 1 [file Image_1.JPEG]

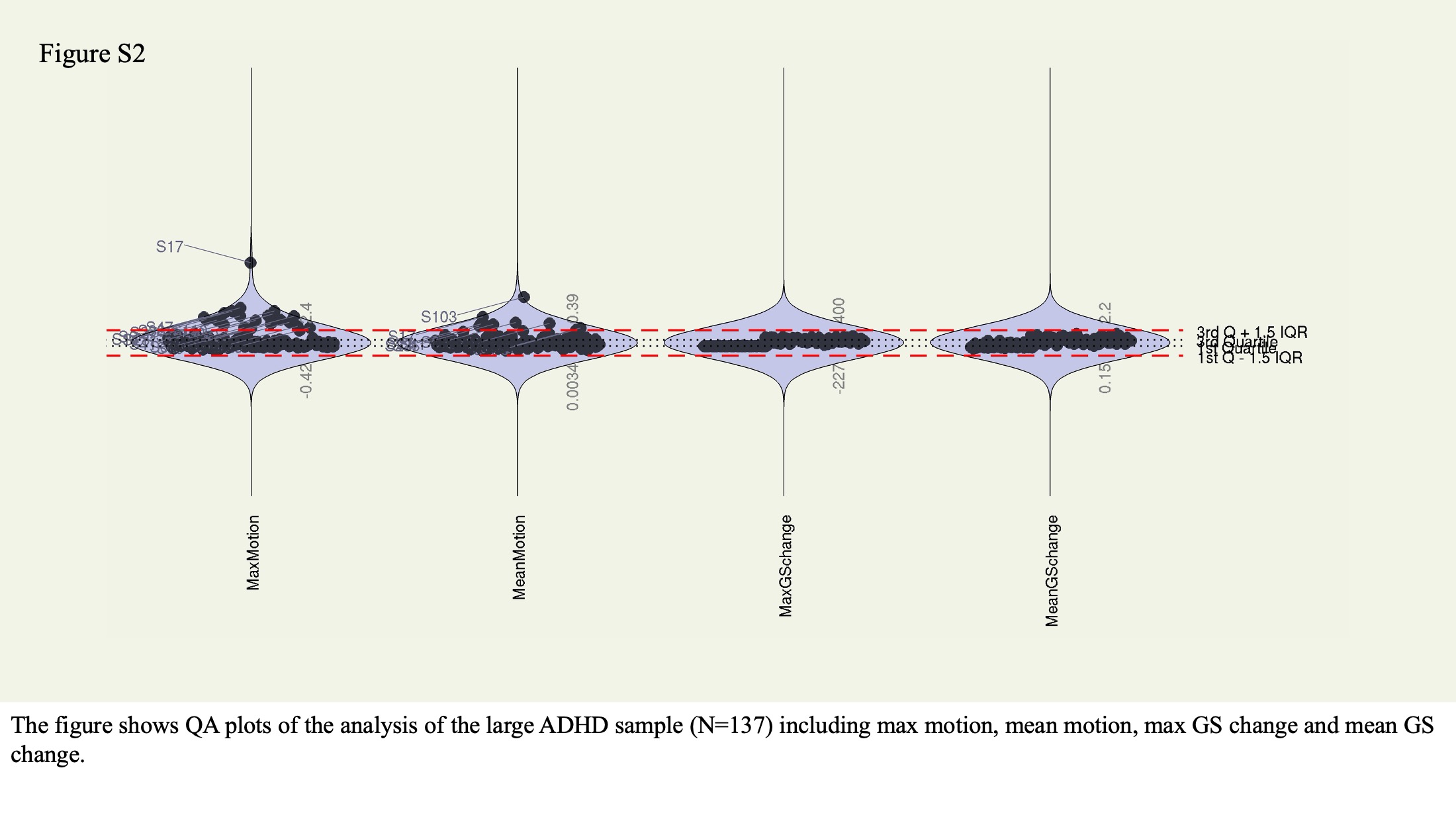

Supplement: Supplementary file 2 [file Image_2.JPEG]

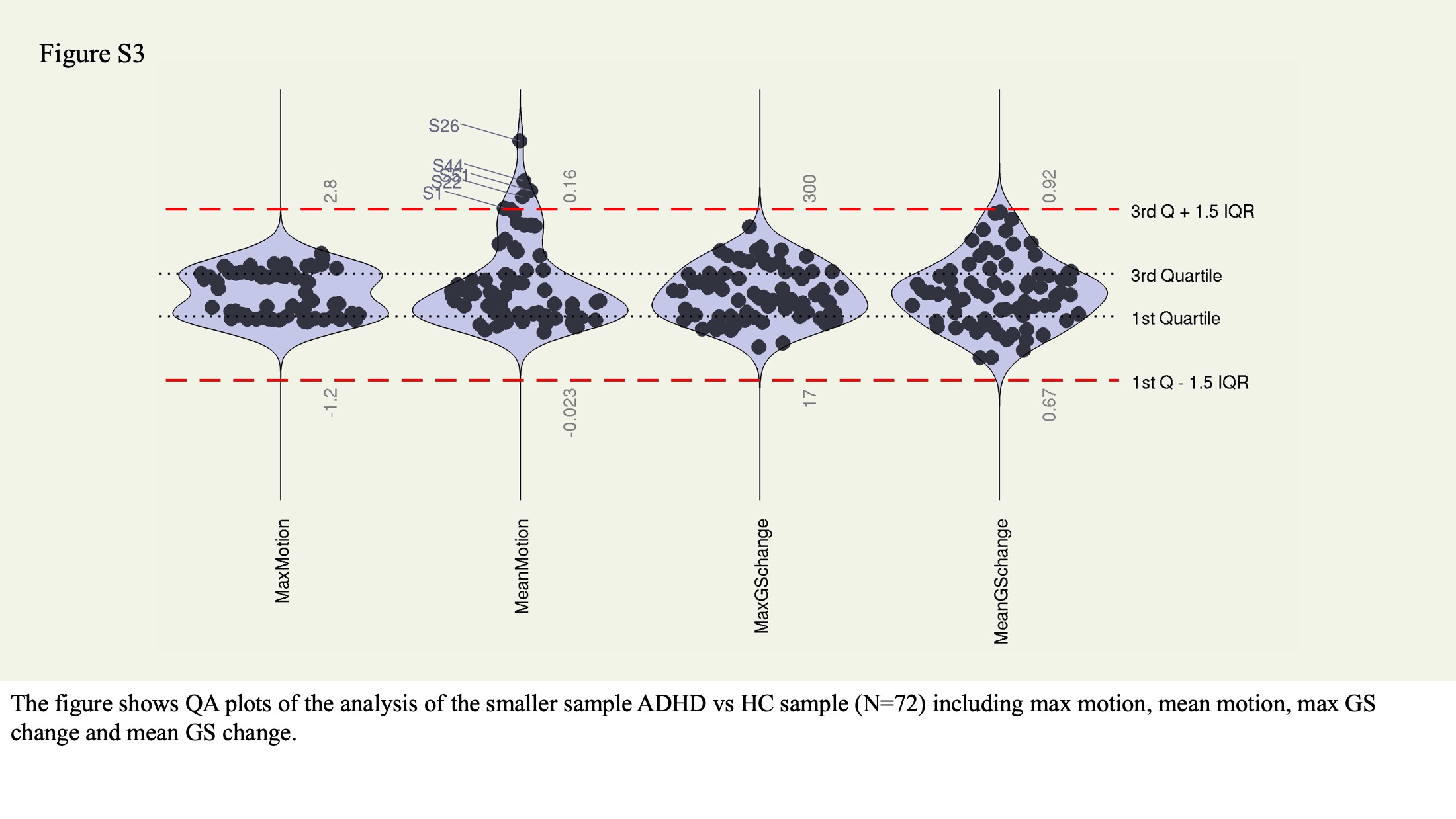

Supplement: Supplementary file 3 [file Image_3.JPEG]
